# MENOPAUSAL SYMPTOM EXPERIENCE OF MID-LIFE WOMEN FAMILY CAREGIVERS OF PERSONS LIVING WITH ALZHEIMER’S DISEASE

**DOI:** 10.1093/geroni/igad104.2339

**Published:** 2023-12-21

**Authors:** Hyein Kim, Heejung Kim, Glenna Brewster, Wonshik Chee, Eun-Ok Im

**Affiliations:** Yonsei University, Seoul, Republic of Korea; Yonsei University, Seoul, Republic of Korea; Emory University, Atlanta, Georgia, United States; Emory University, Atlanta, Georgia, United States; Emory University, Atlanta, Georgia, United States

## Abstract

Midlife women experience a wide range of menopausal symptoms. Such symptoms may be different in midlife women who are family caregivers of persons living with chronic disease, particularly Alzheimer’s disease. Because the cultural stigma of dementia differs among racial/ethnic minority groups, midlife minority women tend to feel burdened with family caregiving and report negative health outcomes compared with White women. This study aimed to explore the racial/ethnic differences in menopausal symptom experienced by midlife women who were family caregivers of persons living with Alzheimer’s disease (MWPLAD). A secondary data analysis was conducted using the data of a cross-sectional online survey among 172 MWPLAD. Self-report questionnaires including the Midlife Women’s Symptom Index were used to gather the data on the women’s sociodemographic characteristics, health-related information, and menopausal symptom experience. Descriptive statistics, one-way analysis of variance and multiple linear regression were adopted for the data analysis. There were significant racial/ethnic differences in midlife women’s symptoms in the total scores (total frequencies and severity scores) and individual sub-scale scores. Psychological symptoms were reported to be more prevalent in Asian MWPLAD than in other racial/ethnic groups. After controlling for race/ethnicity, several factors were associated with the menopausal symptoms experienced by MWPLAD, such as household income, history of obstetrics and gynecology diseases, underweight, and restriction to perform daily activities. Our study findings support the racial/ethnic differences in menopausal symptoms experienced by MWPLAD. More culturally competent interventions should be established considering the significant factors that differently affect the women’s menopausal symptom experience in different racial/ethnic groups.

